# The hypomethylating agent decitabine prior to chemotherapy improves the therapy efficacy in refractory/relapsed acute myeloid leukemia patients

**DOI:** 10.18632/oncotarget.5600

**Published:** 2015-09-10

**Authors:** Xuejie Jiang, Zhixiang Wang, Bingjie Ding, Changxin Yin, Qingxiu Zhong, Bing Z. Carter, Guopan Yu, Ling Jiang, Jieyu Ye, Min Dai, Yu Zhang, Shuang Liang, Qingxia Zhao, Qifa Liu, Fanyi Meng

**Affiliations:** ^1^ Department of Hematology, Nanfang Hospital, Southern Medical University, Guangzhou, China; ^2^ Hematopathy Diagnosis and Therapy Center, Kanghua Hospital, Dongguan, China; ^3^ Section of Molecular Hematology and Therapy, Department of Leukemia, The University of Texas MD Anderson Cancer Center, Houston, TX, USA; ^4^ Southern Medical University, Guangzhou, China; ^5^ Department of Stomatology, Nanfang Hospital, Southern Medical University, Guangzhou, China

**Keywords:** hypomenthylating agent, decitabine, refractory, relapse, AML

## Abstract

In this study, we investigated the effect of pre-treatment with demethylating agent decitabine on susceptibility to chemotherapeutic drugs in HL60/ADR, Kasumi-1 and primary AML cells. Cytotoxic effect was increased by decitabine through activation of p53 and inhibition of c-Myc, Survivin and Bcl-2. We demonstrated in clinic that combination of decitabine and HAA consisting of harringtonine, aclarubicin and cytarabine was effective and safe to treat patients with refractory, relapsed or high-risk AML. Decitabine prior to HAA regimen improved the first induction complete response rate, and significantly prolonged overall survival and disease-free survival in these patients compared with HAA alone. These findings support clinic protocols based on decitabine prior to chemotherapy to overcome resistance and improve therapeutic efficacy in AML patients.

## INTRODUCTION

Most acute myeloid leukemia (AML) patients with adverse prognosis either fail to achieve complete remission (CR) or relapse after short-term remission, such as patients with complex karyotype, FLT3 mutiation, myelodysplasia-related changes, therapy-related AML (t-AML) [1], therapeutic options for these patients are limited. New strategies are needed to improve therapeutic effect in these patients. Gene silencing via DNA methylation are frequent and reversible in high-risk AML [2, 3], it provides the possibility to treat AML patients with DNA-hypomethylating agent.

Hypomethylating agent decitabine is activated intracellularly by deoxycytidine kinase, the active metabolite 5-aza-2′-deoxycytidine-triphosphate inhibits methyltransferase after incorporation into DNA [4]. DNA hypomethylation leads to restore function of methylated gene involved in cellular differentiation and apoptosis [5]. Overexpression of DNA methyltransferase DNMT3B is a predictor of poor survival in AML, these patients may benefit from demethylating agent [6, 7]. Decitabine alone has limited anti-leukemia activity and minimal extramedullary toxicity [8, 9]. It was reported that 15.7% of AML patients achieved CR after single decitabine treatment, and the median OS was 177 days [4]. Synergistic cytotoxicity had been observed in AML cells treated with cytotoxic drug and decitabine [10–12]. Limited studies demonstrated that epigenetic priming with decitabine prior to chemotherapy was likely to increase efficacy in AML patients with unfavorable-risk factors [13]. However, methylated genes weren't definite, effect of decitabine prior to chemotherapy hasn't been well evaluated in clinic.

In this study, we investigated the effect of hypomethylating on susceptibility to cytotoxic drugs in chemoresistant AML cells. Efficacy and safety of decitabine prior to HAA regimen was evaluated in refractory, relapsed and high-risk AML patients.

## RESULTS

### Decitabine improves cytotoxicity of drugs in chemoresistant AML cells

**Figure 1:**
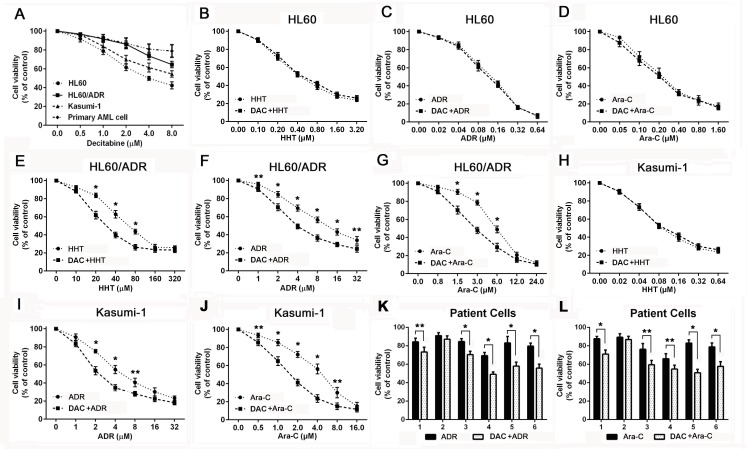
Decitabine increased sensitivity to cytotoxic drugs in chemoresistant AML cells Viability was measured by MTT assay in AML cells and patient samples Different AML cells **A.** were treated with decitabine for 72h. HL60 **B.**, **C.** and **D.**, HL60/ADR **E.**, **F.** and **G.** and Kasumi-1 **H.**, **I.** and **J.** were treated with different concentrations of harringtonine, adriamycin or cytarabine for 24h. Primary AML cells **K.** and **L.** were treated with 10μM adriamycin or cytarabine for 24h. (*P < 0.01, **P < 0.05).

Chemoresistant AML cell lines and primary cells (*n* = 6, Table 1) were treated with different concentrations of decitabine for 72h. Dose-response curves were shown (Figure 1A). Based on growth inhibition assay, 1μM decitabine was used in the subsequent experiments. Sensitivity to harringtonine, adriamycin or cytarabine was evaluated in AML cells after decitabine pretreatment. There was no difference in sensitivity to each drug in HL60 cells (Figure 1B, 1C and 1D). Decitabine increased sensitivity to harringtonine, adriamycin and cytarabine in HL60/ADR cells (Figure 1E, 1F and 1G). Cytotoxicity of these drugs except harringtonine was enhanced in Kasumi-1 cells (Figure 1H, 1I and 1J). Moreover, effects of adriamycin and cytarabine were also increased by decitabine in primary samples from refractory AML patients (Figure 1K and 1L). These data demonstrated that cytotoxicity of drugs was increased by decitabine in chemoresistant AML cells.

**Table 1:** Characteristics of patients with AML (n=6)

No	Age	FAB	Karyotype	FLT3/Kit	Disease status	BM Blast (%)
1	56	M4	46XY	WT/WT	Refractory	72
2	36	M2	complex	ITD/WT	high-risk	87
3	19	M5	46XX	N/A N/A	Relapse	92
4	42	M2	t(8,21)	WT/Mut	Refractory	74
5	49	M1	N/A	N/A N/A	Refractory	79
6	30	M5	46XY	WT/WT	Relapse	80

Abbreviation: FAB. French-American-British classification system; WT. Wild type; N/A. not available; ITD. FLT3 internal tandem duplication; Mut. Mutation.

### Decitabine increases drug-induced apoptosis in chemoresistant AML cells

**Figure 2:**
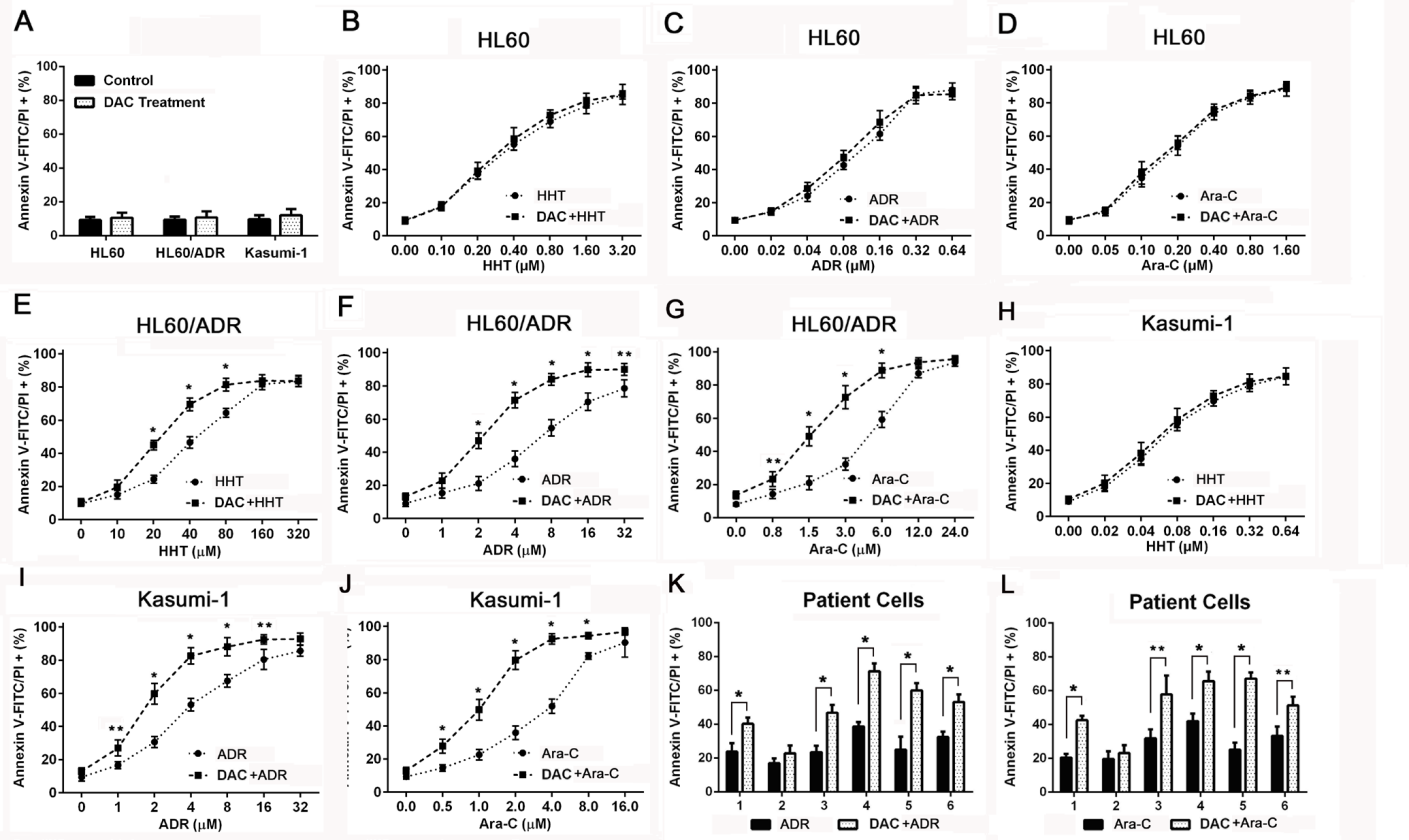
Decitabine enhanced drug induced apoptosis in chemoresistant AML cells Apoptosis was measured by flow cytometry in AML cells after stained with Annexin V-FITC/PI. AML cells **A.** were pretreated with 1μM decitabine for 72h. HL60 **B.**, **C.** and **D.**, HL60/ADR **E.**, **F.** and **G.** and Kasumi-1 **H.**, **I.** and **J.** were treated with different concentrations of harringtonine, adriamycin or cytarabine for 24h. Primary AML cells were treated with 10μM adriamycin (E) or cytarabine for 24h (F). (*P < 0.01, **P < 0.05).

To investigate whether cytotoxicity was related to apoptosis, chemoresistant AML cells and primary AML cells were treated with harringtonine, adriamycin and cytarabine for 24h. Apoptosis was determined by flow cytometry after cells were stained with Annexin V-FITC/PI. Apoptosis proportion was unchanged after 1μM decitabine pretreatment in AML cells (Figure 2A). Decitabine didn't change drug-induced apoptosis in HL60 cells (Figure 2B, 2C and 2D), but increased the apoptosis in HL60/ADR cells (Figure 2E, 2F and 2G). Apoptosis induced by these drugs except harringtonine was enhanced in Kasumi-1 cells (Figure 2H, 2I and 2J). It was also demonstrated that decitabine also increased adriamycin and cytarabine induced apoptosis in most primary AML samples (Figure 2K and 2L). The data showed that decitabine increased drug-induced apoptosis in chemoresistant AML cells.

**Table 2:** Characteristics of patients with AML

Characteristics	Decitabine+HAA	HAA	p-value
	n (range or %)	n (range or %)	
No. of patients	23	24	
Age	36.3(16-59)	39.8 (18-58)	0.672
Male/Female	14/9	16/8	0.766
FAB subtype			
M2	3(13.0)	4(16.7)	1.000
M4	2(8.7)	2(8.3)	1.000
M5	11(47.8)	14(58.3)	0.564
M6	2(8.7)	0(0.0)	0.234
M7	1(4.3)	1(4.2)	1.000
Unclassification	4(17.4)	3(12.5)	1.000
Clinic unfavourable factors			
Therapy-related	3(13.0)	2(8.3)	0.666
Refractory	7(30.4)	10(41.7)	0.547
Relapse	9(39.1)	6(25.0)	0.359
Blasts inhibition<50%	2(8.7)	5(20.8)	0.416
Myelodysplasia-related	2(8.7)	1(4.2)	0.609
Cytogenetic			
Better-risk	1(4.3)	1(4.2)	1.000
Intermediate-risk	14(60.9)	10(41.7)	0.248
Poor-risk	6(26.1)	9(37.5)	0.534
Not available	2(8.7)	4(16.7)	0.666
Molecular biology			
FLT3-ITD+	2(8.7)	3(12.5)	1.000
MLL+	6(26.1)	4(16.7)	0.494
P53 deletion	1(4.3)	2(8.3)	1.000
Others	2(8.7)	1(4.2)	1.000
Negative	10(43.5)	11(45.8)	1.000
Not available	2(8.7)	3(12.5)	1.000

### Decitabine increases effect of HAA in chemoresistant AML cells

**Figure 3:**
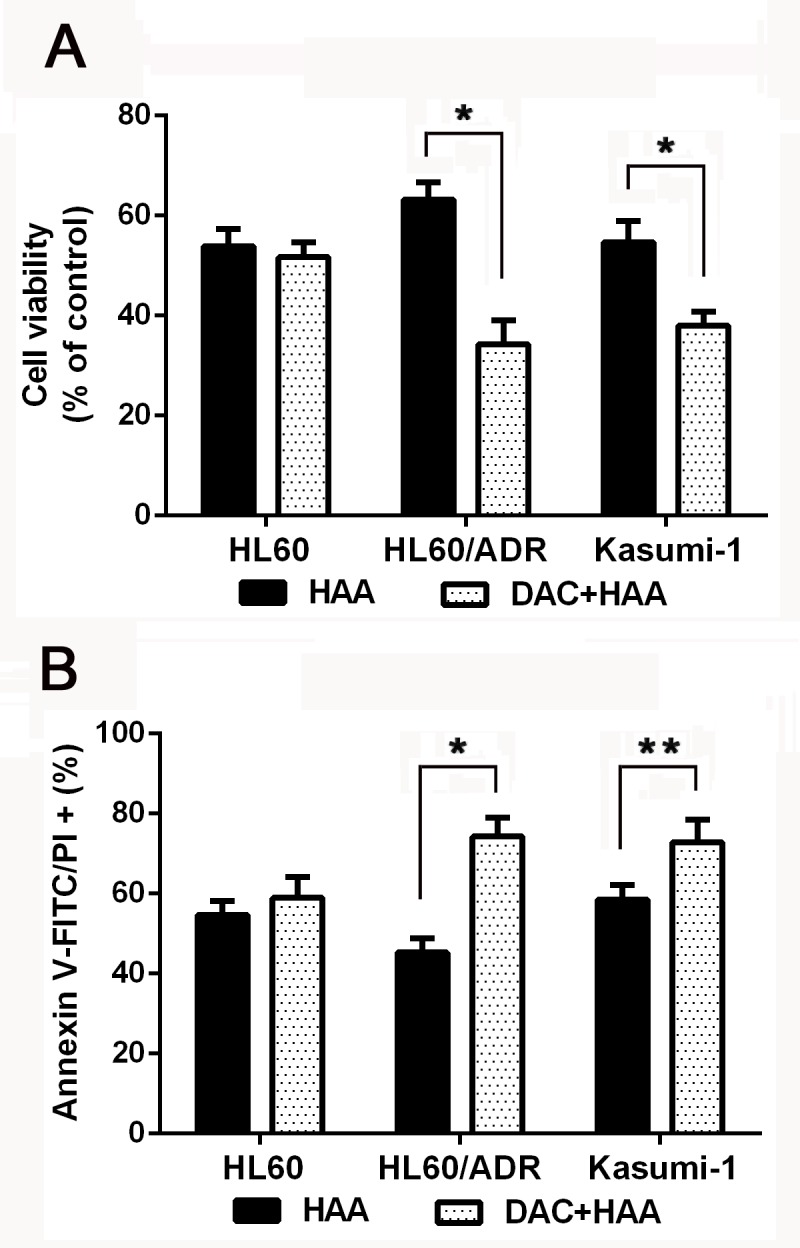
Decitabine increased cytotoxic effect of HAA in chemoresistant AML cells HL60 cells were treated with the mixture of 0.2 μM harringtonine, 0.04μM adriamycin and 0.1μM cytarabine, HL60/ADR cells were treated with the mixture of 20μM harringtonine, 2μM adriamycin and 1.5μM cytarabine, Kasumi-1 cells were treated the mixture of 0.04μM harringtonine, 2μM adriamycin and 1μM cytarabine. Cell viability **A.** and apoptosis **B.** were measured to evaluate the effect of HAA in AML cells. (*P < 0.01, **P < 0.05).

To evaluate effect of decitabine plus HAA regimen on proliferation and apoptosis, HL60, HL60/ADR and Kasumi-1 cells were treated with the mixture of harringtonine, adriamycin and cytarabine for 24h. Cell viability and apoptosis were used to evaluate the sensitivity to drugs. Decitabine didn't change the sensitivity and drugs induced apoptosis in HL60, but increased the cytotoxicity of HAA in HL60/ADR cells and Kasumi-1 cells (Figure 3A and 3B). The data indicated that decitabine increased cytotoxic effect of HAA in chemoresistant AML cells.

### Decitabine induces apoptotic signals

**Figure 4:**
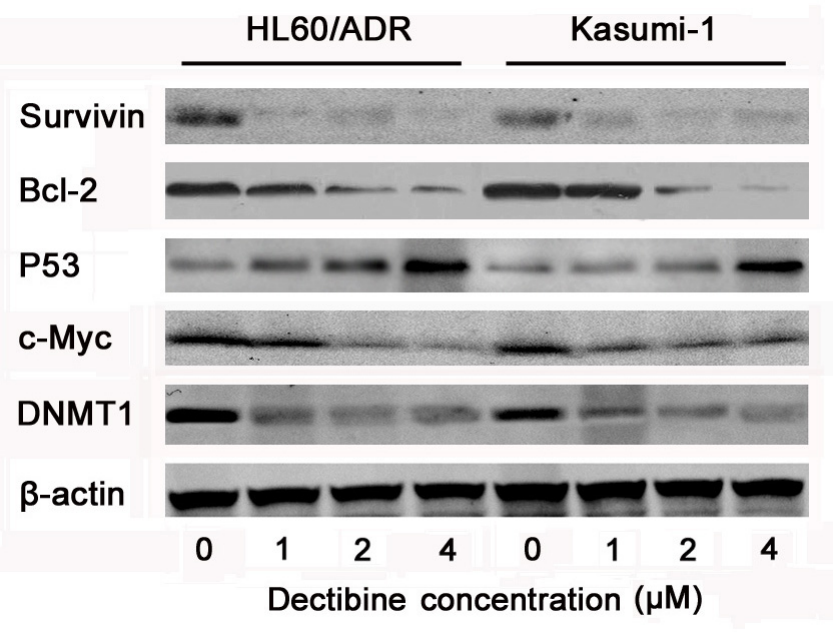
Apoptosis signal pathways were activated by decitabine in chemoresistant AML cells HL60/ADR and kasumi-1 cells were lysed after treated with 0, 1, 2, 4μM decitabine for 72h, and antibodies against β-actin was used as loading control of whole cell proteins in western blotting. The expression of DNMT1, c-Myc, P53, Bcl-2 and Survivin were determined in AML cells.

To confirm that apoptosis contributes to sensitization to cytotoxic drugs, DNA-methyltransferase 1 (DNMT1) and apoptotic proteins were analyzed in HL60/ADR and Kasumi-1 cells (Figure 4). Western blotting showed that decitabine decreased expression of DNMT1 in both cell lines. Transcription factor c-Myc, anti-apoptotic protein Bcl-2 and Survivin were inhibited, and tumor suppressor protein p53 was activated after hypomethylating treatment. These results indicated that apoptotic signals were activated after decitabine treatment in chemoresistant AML cells.

### Efficacy of decitabine prior to chemotherapy

In 23 objects enrolled in decitabine plus HAA, 16 (69.6%) responded to the first course of induction therapy: 14 CR (60.9%) and 2 PR (8.7%). All subjects with PR received the second course induction, 1 of them achieved complete remission. It brought the overall CR was 65.2%. 24 patients were enrolled in HAA without decitabine, 11 (45.8%) responded to the first induction: 7 CR (29.2%) and 4 PR (16.7%), 3 in 4 patients with PR achieved remission after the second induction, and overall CR reached 41.7% after 2 cycles of induction. The CR ratio after first induction in decitabine plus HAA was higher than that in HAA alone (*p* = 0.041). There was also a good tendency in overall CR ratio after combined treatment although there wasn't significance between two groups (Table 3).

**Figure 5:**
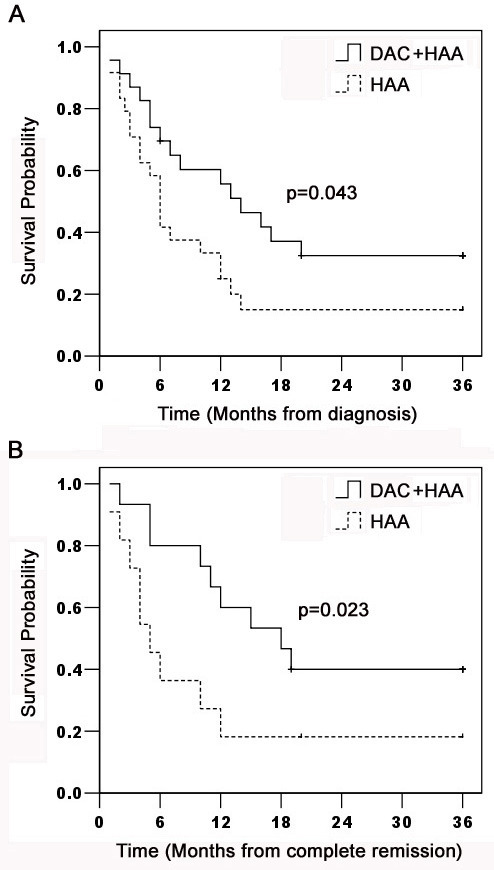
Kaplan-Meier estimation of OS and DFS in AML patients AML Patients were enrolled in decitabine prior to HAA (*n* = 23) or HAA alone (*n* = 24). Patients were followed up to 36 months for OS (*p* = 0.043) and DFS (*p* = 0.023) shown in Figure 5A and 5B.

Patients after CR continued to receive consolidation and intensification chemotherapy alternatively in combination with decitabine. Patients were followed up to 36 months. The median OS was 14 months in decitabine plus chemotherapy, and 6 months in chemotherapy alone. The lower bound of the 95% confidence interval is 8.0 and 4.8 months in each group (Figure 5A). The median DFS were respectively 18 and 5 months in 15 patients treated with decitabine and 10 patients without decitabine, and the lower bound of the 95% confidence interval is 9.2 and 2.6 months in each group (Figure 5B). Patients treated with decitabine had significantly longer OS (34.8%) and DFS (40%) than those without decitabine (Both *p* < 0.05). 6 (40%) patients with decitabine treatment and 2 (20%) patients without decitabine treatment were still alive in CR after 36 months. The data supported that dacitabine improved chemotherapeutic efficacy in refractory/relapsed AML patients.

### Safety of decitabine prior to chemotherapy

Patient's routine blood test was done every one or two days after chemotherapy. Neutrocytopenia and thrombcytopemia were defined when neutrophil count and platelet count were respectively less than 0.5×10^9^/L and 20×10^9^/L. Durations of neutrocytopenia and thrombcytopemia were determined in each cycle of chemotherapy, no significant difference was observed in the time of neutrophil recovery or platelet recovery after induction therapy and intensification therapy (Table 3). The incidences of infection in two groups were also similar after chemotherapy (Table 3). The clinic data indicated that decitabine plus HAA regimen was well-tolerated to treat AML patients.

**Table 3:** Efficacy and safety of Decitabine+HAA treatment

Groups	Decitabine+HAA	HAA	p-value
	n (range or %)	n (range or %)	
CR after first induction	14(60.9)	7(29.2)	0.041
Overall CR after 2 cycle	15(65.2)	10(41.7)	0.147
Induction therapy			
Neu<0.5G/L (d)	18(14-19)	17(13-20)	0.832
Plat<20G/L(d)	8(6-11)	9(5-12)	0.798
Infection accident (n)	19(82.6)	18(75.0)	0.724
Therapy after CR			
Neu<0.5G/L(d)	12(9-15)	10(7-13)	0.631
Plat<20G/L(d)	6(5-9)	7(5-10)	0.544
Infection incidence (n)	10(66.7)	6(60.0)	1.000

## DISCUSSION

Overexpression of DNMT1 contributes to pathogenesis of leukemia [18]. HL60/ADR and Kasumi-1 are multi-drug resistant AML cells with high expression of DNMT1. In this study, we found that decitabine depleted DNMT1, activated apoptotic signal, and improved chemotherapeutic cytotoxicity in HL60/ADR and Kasumi-1 cells. Decitabine prior to HAA regimen improved CR ratio of first induction compared with HAA alone in refractory, relapse or high-risk AML patients, their overall CR rates weren't different after 2 cycles of induction therapy. However, OS and DFS were prolonged in these patients treated by decitabine plus chemotherapy. It was safe to treat AML patients for the same status in myeloid suppression and infection after chemotherapy.

Aberrant DNA methylation leads to epigenetic silencing of genes, it is also correlated with leukemogenesis and disease relapse in AML. Silencing genes are reactivated after hypomethylating treatment, so decitabine is reasonable to improve chemotherapeutic efficacy in AML [19–21]. In our study, it was demonstrated that decitabine increased cytotoxic effect in chemoresistant AML cells via DNA damage-associated P53 activation and inhibition of survivin and Bcl-2. Tumor suppressor p53 inactivation is frequently associated with chemotherapy resistance in AML, and suppress of p53 signaling contributes to the resistance towards apoptosis in leukemia cells [22, 23]. DNA demethylation is an effective therapeutic strategy to restore p53 function, and it leads to p53-correlated inhibition of survivin and Bcl-2 in cancer cells [24–26]. Our data indicated that survivin was inhibited by overexpression of p53 in chemoresistant AML cells treated by decitabine. Proto-oncogene *c-Myc is* aberrantly expressed in many solid tumors and hematological malignancies, it *is* responsible for proliferation, apoptosis and tumorigenesis in cancer cells [27–29]. It was also reported that c-Myc contributed to maintenance of AML stem cells and drug-resistance [30, 31]. Overexpression of c-Myc indicates a poor prognosis in AML patients, its inhibition was also considered as an attractive way to overcome drug resistance [32, 33]. Our results indicated that inhibition of c-Myc increased drug's cytotoxicity after demethylating treatment, it was consistent with previous studies in chronic myeloid leukemia [34].

DNA methylation signature was indentified as a biological distinction and conferred prognostic value in AML [35]. Decitabine was known to reactivate methylated genes, and emerged as an alternative in initial, salvage or consolidation therapy in AML patients [8, 36]. It was also documented that older or relapsed patients with AML benefited from decitabine treatment with a good toleration [4, 37]. But single decitabine may have limited anti-leukemia effect in relapsed/refractory AML, the results from retrospective studies suggested that it is possible for decitabine in combination with chemotherapy to improve efficacy in AML patients with unfavorable factors. A phase I study demonstrated that epigenetic priming with decitabine prior to standard induction was administered in an attempt to improve the response rates in AML [13]. It was recently reported that decitabine in combination with G-CSF, cytarabine and aclarubicin was well-tolerated and efficacy to treat elderly patients with AML [38]. Moreover, clinical outcomes also indicated that combination of decitabine and chemotherapy improved CR rate and OS in refractory AML patients [39, 40]. But there were few evidences in intensified therapy with decitabine in AML.

In our study, AML patients with refractory, relapsed or high-risk factors received induction and consolidation chemotherapy in combination with decitabine. There was a better first induction CR rate in decitabine prior to HAA than HAA alone, as well as a good tendency in overall CR rate. The higher CR rate may be correlated with epigenetic modifier decitabine prior to harringtonine, aclacinomycin and cytarabine. The CR rate of HAA regimen was in agreement with the previous reports in refractory/relapsed AML with unfavourable cytogenetics [41]. AML patients after CR continued to receive chemotherapy combined with decitabine, the better estimated 3-year OS and DFS indicated an encouraging survival in these AML patients. 40% AML patients with decitabine treatment were still alive in CR after 3-year follow-up. Hypomethylating agent had the ability to increase sensitivity to cytotoxic drugs, so decitabine prior to chemotherapy may be used as frontline, salvage and consolidation therapy in high-risk AML patients [36]. One concern about the combination had a risk to prolong duration of myelosuppression. Instead, we found that the time of neutrophil recovery or platelet recovery in chemotherapy combined with decitabine was similar to that in chemotherapy alone. Moreover, no significant difference between two regimens was detected in infection incidence. Our data indicated that decitabine piror to chemotherapy was efficacy and safe to treat refractory/relapsed AML patients. Other clinic trials also demonstrated that chemotherapy alternating with decitabine was well-tolerated and effective in older AML patients [38, 42]. So decitabine prior to chemotherapy may be considered as an alternative therapeutic regimen in refractory AML patients.

In conclusion, our results demonstrated that decitabine increased cytotoxic effect on chemoresistant AML cells via P53 activation and inhibition of c-Myc, survivin and Bcl-2. Decitabine prior to HAA regimen was safe and efficacy to treat refractory/relapsed or high-risk AML. These findings support clinic protocols based on decitabine prior to chemotherapy to overcome resistance and improve therapeutic efficacy in AML patients.

## MATERIALS AND METHODS

### Cell culture

The human AML cell lines HL60, HL60/ADR and Kasumi-1 (Institutes for Biological Sciences Cell Resource Center, Chinese Academy of Sciences, Shanghai, China) were cultured in RPMI-1640 medium (Hyclone, MA, USA) supplemented with 10% heat-inactivated fetal bovine serum (GIBCO, NY, USA) in a humidified atmosphere of 5% CO_2_ at 37°C. HL60/ADR displayed 79-fold higher resistance to adriamycin than the parental line HL60, and it was maintained with 0.1μM adriamycin (Doxorubicin) (Sigma, MO, USA) to ensure its drug resistance. Six bone marrow samples were obtained from patients with refractory, relapsed or high-risk AML except M3 (Table 1), those patients failed to achieve remission following at least 2 induction chemotherapy, those recurred after complete remission (<5% marrow blasts) and resisted to the re-induction protocol, those had complex karyotype or FLT3-ITD mutation, or AML patients with t(8;21)(q22;q22) and c-Kit mutation [14]. Leukemia blasts in bone marrow were over 70% in these AML patients. Mononuclear cells were isolated by Ficoll separation and suspended in α-MEM medium with 20% fetal bovine serum. The human subject study was conducted under the institutional ethics committee's approval of Nanfang Hospital, Southern Medical University, and written informed consent was obtained from the patients.

### Decitabine treatment

To determine decitabine dose-response curves, HL60, HL60/ADR, and Kasumi-1 cells at density of 1×10^5^cells/ml were treated with various concentrations of decitabine (Sigma Aldrich, MO, USA) for 3 days. Viable cells were counted using trypan blue exclusion. HL60, HL60/ADR, Kasumi-1, and primary AML cells were treated with 1.0 μM decitabine for 72 hours and fresh decitabine was added daily. Treated cells were collected and used in the following experiments.

### Drug sensitivity assay

Sensitivities to harringtonine (HHT), adriamycin (ADR) and cytarabine (Ara-C) were determined using 3-(4,5-Dimethylthiazol-2-yl)-2,5-diphenyltetrazolium bromide (MTT) (Sigma, MO, USA) in AML cells. Briefly, After cells were seeded into 96-well plate, different concentrations of harringtonine, adriamycin, cytarabine or their combination were subsequently added and incubated for 24h. 20μL MTT solution (5mg/mL) was added in 200μL media plus drug in each well for the last 4h of 24h culture. Absorbance was measured at 540nm in a spectrophotometer (Thermo Scientific Evolution 600, China), and cell viability was calculated. Dose-response curves were measured to evaluate cytotoxic effect.

### Apoptosis assessment

HL60, HL60/ADR and Kasumi-1 cells were treated with different concentrations of harringtonine, adriamycin, cytarabine or their combination for 24h, as well as primary AML cells. The treated cells were collected and stained with Annexin V-FITC/PI (NanJing KeyGen Biotechnology, NanJing, China), and subjected to BD flow cytometry (Becton Dickinson, CA, USA) for apoptosis analysis.

### Immunoblot analysis

HL60/ADR, Kasumi-1 cells were treated with 1.0, 2.0, 4.0μM decitabine for 72h. Cells were harvested and lysed in cold lysis buffer (0.5 M Tris-HCl, pH 6.8, 2 mM EDTA, 10% glycerol, 2% SDS, 5% β-ercaptoethanol) containing 0.5mM dithiothreitol (DTT) and 0.1mM phenylmethylsulfonyl fluoride (PMSF), the supernatant was collected after centrifugation (12000rpm, 10min). The protein concentration was determined using BCA protein determination kit (Pierce Chemical, Rockford, IL, USA). Cell extracts were subjected to sodium dodecylsulfate-polyacrylamide gel electrophoresis (SDS-PAGE), and transferred to polyvinylidene difluoride (PVDF) membrane. The membranes were blocked with 5% milk in Tris-buffered saline containing 0.1% Tween 20, and immunoblotted overnight at 4°C with the appropriate primary antibody, followed by treatment with horseradish peroxidase-linked secondary antibody. The immunocomplex was visualized using a chemiluminescence phototope-horseradish peroxidase kit. Antibody against β-actin (CST, MA, USA) was used to ensure equivalent loading of whole cell protein.

### Patients and treatment

#### Patients eligibility

AML Patients with unfavorable factors by World Health Organization (WHO) criteria were eligible for this study (Table 2) [15]. Twenty-three patients were enrolled in decitabine prior to HAA regimen from 2011 to 2014. They had different unfavorable characteristics with median age 36.3 (range from 16 to 59). Among them, Six patients had poor-risk cytogenetic, two was patents with FLT3-ITD mutation, six was patents with MLL rearrangement, two were AML with myelodysplasia-related changes, three were therapy-related AML, nine were relapsed patients, and two were patients with first induction therapy resulting in inhibiting no more than 50% of blasts. Twenty-four patients were enrolled in HAA without decitabine regimen with median age 39.8 (range from 18 to 58). Among them, nine patients had poor-risk cytogenetic, three was patents with FLT3-ITD mutation, four was patents with MLL rearrangement, one was AML with myelodysplasia-related changes, two were therapy-related AML, six were relapsed patients, and five were patients without inhibiting 50% of blasts after first induction therapy. Other characteristics of patients were shown in Table 2. Additional inclusion criteria were as the following: Eastern Cooperative Oncology Group (ECOG) performance status of 0 to 2; normal renal and hepatic function; life expectancy greater than 12 weeks; and ineligibility for endure induction chemotherapy. Exclusion criteria included the following: diagnosis of acute promyelocytic leukemia; good-risk cytogenetics as defined by Southwest Oncology Group (SWOG) criteria [16]; CNS leukemia; prior therapy without decitabine and uncontrolled infection.

Patients in decitabine plus HAA regimen were treated in department of hematology in Nanfang Hospital, Southern Medical University, Guangzhou, China. This study protocol was approved by the Ethics Committee of Nanfang Hospital. Written informed consents were obtained from the patients prior to the study.

#### Treatment

Patients in study group were subjected to infusional decitabine (20mg/m^2^ for 3 days) before HAA regimen using infusional harringtonine (2.5mg/m^2^ daily for 3 days, generally the first 3 days of aclarubicin and cytarabine) with aclarubicin (12mg/m^2^ daily for 7 days) and cytarabine (100mg/m^2^ daily for 7 days). Patients in control group were treated with HAA alone. Patients received supportive care as clinically indicated, including blood transfusion, growth factor injected hypodermically, standard antiemetic and antimicrobial therapy. Each cycle was repeated every 4 weeks.

Subjects received subsequent therapy based on efficacy after induction cycle. Patients with partial remission (PR) were re-induced by decitabine plus HAA, regular assessment was performed as before. All patients with complete remission (CR) received one cycle of consolidation with the same regimen as induction therapy, then followed by four cycles of intensification therapy alternatively using decitabine (20mg/m^2^ for 3 days) prior to FA regimen (35mg/m^2^ fludarabine combined with 1.5g/m^2^ cytarabine daily for 5 days) or high-dose (HD) Ara-C (2g/m^2^ cytarabine daily for 3 days). Patients in HAA group received the same intensification therapy without decitabine. After one or two induction cycles, patients without achieving CR switched to other therapeutic projects or allogenic stem cell transplantation therapy at the discretion of their treating physician.

#### Efficacy and side-effect assessment

Blood routine, liver and kidney function tests, chest X-ray, cardiac enzymes and electrocardiograms were performed before and after treatment. Bone marrow aspirates were performed and evaluated by local hematopathologists after each cycle. Therapeutic efficacy was evaluated according to International Working Group Criteria [17]. Patients with CR required normalization of peripheral blood counts with less than 5% blasts in bone marrow. Patients with PR required all of the hematologic values for CR but with a decrease of at least 50% in the percentage of blasts to 5-25% blasts in the bone marrow. Responsive patients were identified as patients achieving CR or PR after induction therapy. Side-effects of chemotherapy were graded according to WHO criteria of grades 0 to IV. Patients had been followed up to 36 months, overall survival (OS, defined as the interval between the date of initial treatment and the date of last follow-up or death) and disease-free survival (DFS, time from first CR to leukemia relapse) were calculated and used in compared analysis.

### Statistical analysis

Data from *in vitro* study were reported as mean ± standard deviation, and statistical significance of difference between groups was determined by two-sided student's t-test. In clinic study, the primary end point was morphologic CR, secondary end points included OS and DFS. CR rate was calculated along with the 95% CI, and analyzed using the Chi-square test. Kaplan-Meier product-limit estimator was used to describe OS and DFS. Cumulative incidence of relapse was used to describe remission duration, and death was a competing risk factor. Statistical significance was evaluated at the 0.05 alpha level. All analyses were performed using SPSS software 22.0.
